# ATOH8, a regulator of skeletal myogenesis in the hypaxial myotome of the trunk

**DOI:** 10.1007/s00418-013-1155-0

**Published:** 2013-11-02

**Authors:** Ajeesh Balakrishnan-Renuka, Gabriela Morosan-Puopolo, Faisal Yusuf, Aisha Abduelmula, Jingchen Chen, Georg Zoidl, Susanne Philippi, Fangping Dai, Beate Brand-Saberi

**Affiliations:** 1 Department of Anatomy and Molecular Embryology, Institute of Anatomy, Ruhr University Bochum, 44801 Bochum, Germany; 2 Faculty of Biology, University of Freiburg, Schänzlestr. 1, 79104 Freiburg, Germany; 3 Department of Molecular Embryology, Institute of Anatomy and Cell Biology, University of Freiburg (Albert-Ludwigs University), Albertstrasse 17, 79104 Freiburg, Germany; 4 Groupe Hospitalier Pitié-Salpetrière, Institut de Myologie, Université Pierre et Marie Curie/Paris 6/Inserm UMR_S 974, CNRS UMR 7215, Paris, France; 5 Department of Neuroanatomy and Molecular Brain Research, Ruhr University Bochum, 44801 Bochum, Germany; 6 Present Address: Medizinische Klinik III, Universitätsklinikum Bergmannsheil, Bürkle-de-la-Camp-Platz 1, 44789 Bochum, Germany; 7 Present Address: Department of Craniofacial Development, King’s College London, Guy’s Campus, Tower Floor 27, London, SE1 9RT UK; 8 Present Address: Department of Molecular and Cellular Neuroscience, York University, Toronto, Canada; 9 Present Address: Neuroonkologie, Neurozentrum, Uniklinikum Freiburg, Hugstetterstr. 49, 79095 Freiburg, Germany; 10 Present Address: Department of Anatomy and Molecular Embryology, Institute of Anatomy, Ruhr University Bochum, 44780 Bochum, Germany

**Keywords:** ATOH8, Myogenesis, C2C12, Chicken embryo, Hypaxial myotome, Trunk

## Abstract

The embryonic muscles of the axial skeleton and limbs take their origin from the dermomyotomes of the somites. During embryonic myogenesis, muscle precursors delaminate from the dermomyotome giving rise to the hypaxial and epaxial myotome. Mutant studies for myogenic regulatory factors have shown that the development of the hypaxial myotome differs from the formation of the epaxial myotome and that the development of the hypaxial myotome depends on the latter within the trunk region. The transcriptional networks that regulate the transition of proliferative dermomyotomal cells into the predominantly post-mitotic hypaxial myotome, as well as the eventual patterning of the myotome, are not fully understood. Similar transitions occurring during the development of the neural system have been shown to be controlled by the Atonal family of helix-loop-helix transcription factors. Here, we demonstrate that *ATOH8*, a member of the Atonal family, is expressed in a subset of embryonic muscle cells in the dermomyotome and myotome. Using the RNAi approach, we show that loss of *ATOH8* in the lateral somites at the trunk level results in a blockage of differentiation and thus causes cells to be maintained in a predetermined state. Furthermore, we show that *ATOH8* is also expressed in cultured C2C12 mouse myoblasts and becomes dramatically downregulated during their differentiation. We propose that ATOH8 plays a role during the transition of myoblasts from the proliferative phase to the differentiation phase and in the regulation of myogenesis in the hypaxial myotome of the trunk.

**Electronic supplementary material:**

The online version of this article (doi:10.1007/s00418-013-1155-0) contains supplementary material, which is available to authorized users.

## Introduction

Skeletal myogenesis during embryonic development is a highly regulated process. It relies on small populations of specified precursor cells that expand and generate committed cells, which ultimately differentiate into myocytes. A balance between the committed and the differentiated state of the myogenic precursors needs to be achieved. This would allow for the formation of a functional tissue and the maintenance of a sufficient population of precursor cells for subsequent phases of growth or tissue repair. A better understanding of how this balance is maintained and regulated during myogenesis would not only enable us to understand how muscle is formed and maintained during development, but may also be significant in the tailoring of therapeutic approaches for the treatment of muscle diseases.

Myotome formation involves an ingression of dermomyotomal myogenic precursors into the myotome from all borders of the dermomyotome. The most recent view on myotome formation proposes that myotomal growth is initiated at the dorsomedial lip of the dermomyotome (DML), which then provides myoblasts that act as a scaffold for later waves of myotomal growth. This theory supports active cellular migration into the myotomal compartment with considerable myocyte contributions from all borders of the somite (Cinnamon et al. 1999; Kahane et al. 2002). Using improved cell lineage and imaging techniques, it was shown that the DML and the ventrolateral lip (VLL) contribute exclusively to the epaxial and hypaxial myotome, respectively. Only incremental growth was shown to occur at the DML and VLL, while myocytes from the rostral and caudal borders contribute to coherent myotomal growth (Gros et al. 2004).

It has been shown that the spatial gene expression of dermomyotomal markers is maintained in the myotome following central dermomyotome dissociation. The cellular identity of the medial and lateral dermomyotome is transferred to the epaxial and hypaxial myotome, respectively (Ahmed et al. 2006). As the VLL contributes exclusively to the hypaxial myotome and the DML solely to the epaxial myotome, it is not surprising that the epaxial myotome growth and patterning is different from that of the hypaxial myotome. Elaborate studies in mutant mice deficient in myogenic regulatory factors have shown that *Myf5* is sufficient for the induction and progression of epaxial myogenesis in the absence of *MyoD*. However, *Myf5* is unable to efficiently rescue the delayed and defective hypaxial myogenesis that occurs in the absence of *MyoD* (Kablar et al. 1997, 1998). Based on the gene expression profile and experimental evidence, the epaxial myotome is not only believed to be more mature than the hypaxial myotome, it is also required for the proper differentiation and patterning of the hypaxial myotome at the trunk level (Kahane et al. 2007). It is speculated that BMP signaling from the lateral plate mesoderm delays myogenic differentiation in the hypaxial myotome (Kahane et al. 2007); however, the intracellular factors that maintain and regulate this delay during hypaxial myotome differentiation are not known.

Our understanding of the mechanism that controls the progression of myogenic precursors to commit to terminal differentiation in both the developmental and regeneration context has greatly improved (reviewed in Bentzinger et al. 2012). On the other hand, the transcriptional networks controlling these fate decisions are still largely unknown. This is not the case in neurogenesis, where the differentiation fate decisions are better described. Work pioneered in *Drosophila* has led to the identification of a family of transcription factors that control the decision of neural cells to embark on lineage commitment and differentiation. These belong to the Atonal family of helix-loop-helix transcription factors. ATOH8 is a novel member of this family (Jarman et al. 1993; Ledent et al. 2002). In mouse, ATOH8 has been found to contribute to endocrine differentiation by modulating specific aspects of Neurog3 function (Lynn et al. 2008). Detailed examination of *ATOH8* shows significant variation in both gene structure and regulatory elements among animals, suggesting a diversification in function (Chen et al. 2011). The murine homolog of ATOH8, Math6, is implicated in the specification and differentiation of cell lineages in the nervous system (Inoue et al. 2001). It has been found that ATOH8 is required for the development of the retina, skeletal muscle, and heart in zebrafish (Yao et al. 2010; Rawnsley et al. 2013). Somitic expression of ATOH8 in the mouse embryo at embryonic stages E9.5 and E12.5 has also been reported (Rawnsley et al. 2013). Our findings further our understanding of the role of ATOH8 in embryonic myogenesis.

We report that the homolog of Atonal, *ATOH8,* is expressed in both developing embryonic muscle and cultured mouse myoblasts (C2C12). We show that during development, ATOH8 is involved in the transition from a progenitor to the differentiated myogenic fate in the hypaxial myotome of the interlimb region. We propose that ATOH8 is required for the fine-tuning of myogenic differentiation during embryonic hypaxial myotome formation.

## Results

### Embryonic expression of *ATOH8* during avian development

**Fig. 1:**
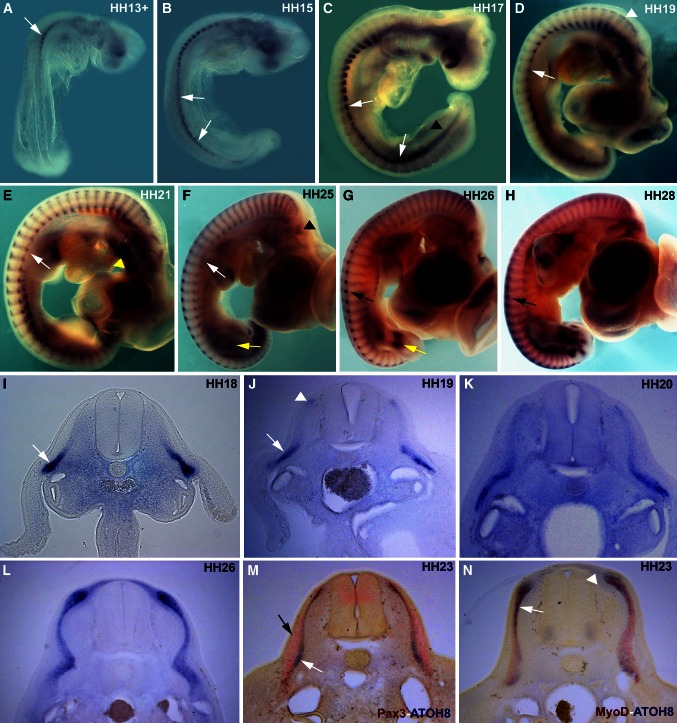
**a**–**h** Whole-mount in situ hybridization expression pattern of *ATOH8* in chicken embryos from HH stage 13–28. Somitic expression of *ATOH8* is shown here at HH13 in the cranial somites (*white arrow* in **a**). At HH15, the expression extends caudally (*white arrows* in **a**). At later stages (HH17), the somitic expression intensifies caudally and medially (*white arrows* in **c**). Note the weak expression of *ATOH8* in the limb level hypaxial myotome in comparison with the cervical and interlimb regions of the trunk (*white arrows* in **d**–**f**). At HH 19, *ATOH8* is detectable in the dorsomedial lip of cranial somites (*white arrowhead* in **d**). At HH21–25, the expression is uniform in the entire myotome. From HH26 to HH28, the medial somitic expression is stronger than in the lateral somites (*black arrows* in **g**, **h**). Expression is also seen in the branchial arches (*yellow arrowhead* in **e**), segmental plate (*black arrowhead* in **c**), otic placode (*black arrowhead* in **f**), and limbs (*yellow arrows* in **f**, **g**). **i**–**n** Vibratome sections of in situ hybridized chicken embryos for *ATOH8* expression from HH18 to HH26. **m**, **n** Double in situ hybridization of *ATOH8* (*blue*)/*Pax3* (*red*) and *ATOH8* (*blue*)/*MyoD* (*red*), respectively, at HH23. *ATOH8* is expressed in the lateral myotome and the lateral lip of the dermomyotome at HH18 and HH19 (*white arrows* in **i**, **j**). The initiation of the medial somitic expression can be seen at HH19 (*white arrowheads* in **j**). At HH20, *ATOH8* is expressed over the entire medio-lateral extent of the myotome. Double in situ hybridization with *Pax3* shows that *ATOH8* is predominantly expressed in the myotome (*white arrow* in **m**), while *Pax3* is predominantly expressed in the dermomyotome (*black arrow* in **m**) and in the intermediate zone of the myotome underlying the central dermomyotome. *ATOH8* expression in the myotome (*white arrows* in **n**) is overlapping that of *MyoD*; however, the medially located subectodermal *ATOH8* expression domain is *MyoD* negative (*white arrowheads* in **n**)

**Fig. 2:**
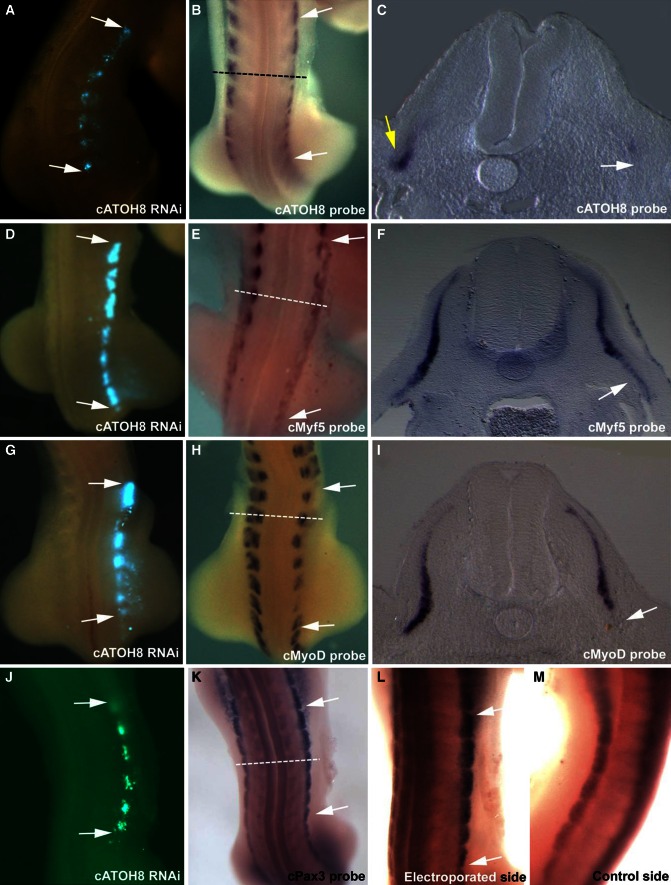
Silencing *ATOH8* in the somites affects the expression of myogenic markers. *ATOH8* was silenced using an *ATOH8*-specific shRNA-EGFP construct targeted to the lateral somite, after which the embryos were reincubated for 24 h. Upon fixation, these embryos were processed for in situ hybridization (*ATOH8*, *Pax3*, *MyoD,* and *Myf5*). The whole-mount images and section images are of the same embryo. The targeted region is indicated by the EGFP expression in the same embryo (*white arrows* in **a**, **d**, **g**, **j**). *ATOH8* is specifically silenced in the region of the shRNA treatment (*white arrows* in **b**, **c**). The *yellow arrow* in **c** shows the normal expression of *ATOH8* at the lateral lip of the dermomyotome on the contralateral side. *ATOH8* silencing leads to a downregulation of *Myf5* (*white arrows* in **e**, **f**) and *MyoD* (*white arrows* in **h**, **i**), whereas *Pax3* (*white arrows* in **k**, **l**) is upregulated. In contrast, the contralateral side of the same embryo does not show any change in *Pax3* expression (**m**). The *dotted lines* in **b**, **e**, **h**, and **k** represent the planes of cross-section shown in **c**, **f**, **i**, and Fig. 3c, respectively. The control EGFP reporter plasmid electroporations did not affect the normal expression pattern of any of the genes tested (Supplementary figure 1 A–H)

The somitic expression of *ATOH8* was first detected at HH10, which thereafter rapidly intensified but maintained its strongest expression in the cranially located somites. From stage HH13 through HH18, *ATOH8* transcripts were detected in the lateral regions of the somites (Fig. 1a–c, i). A detailed analysis revealed that the expression domain was confined to the hypaxial myotome and the lateral lip of the dermomyotome (Figs. 1i–k, 2c; Supplementary fig. 1 I, J). *ATOH8* transcripts in the hypaxial myotome are expressed prominently at the interlimb level, but faded away at the axial levels where the limb buds are present. Moving from stage HH17 toward HH19, the lateral somitic expression domain spreads medially into the myotome, accompanied by a progressive extension of *ATOH8* expression into the more caudally located somites (Fig. 1c, d). The earliest transcripts in the medial myotome and dorsomedial dermomyotomal lip were detected from stage HH19 onward (Fig. 1d, j).

Between stages HH21-26, somitic expression of *ATOH8* was found throughout the entirety of the myotome (Fig. 1e–g, k, l). At HH26, *ATOH8* transcripts were also detected in the subectodermal mesenchyme overlying the neural tube, adjacent to the medial lip of the dermomyotome (Fig. 1g, l). The myotomal expression was still detected at HH28-HH29 (Fig. 1h), but was no longer found by HH30 (data not shown). Double staining showed some overlapping with the *MyoD* expression domain in the myotome (Fig. 1n), while *Pax3* was expressed in the dermomyotome and in the intermediate domain (Ben-Yair and Kalcheim 2005) corresponding to the delaminating central dermomyotome (Fig. 1m).

### The effect of *ATOH8* silencing on myogenic markers in the somite

**Fig. 3:**
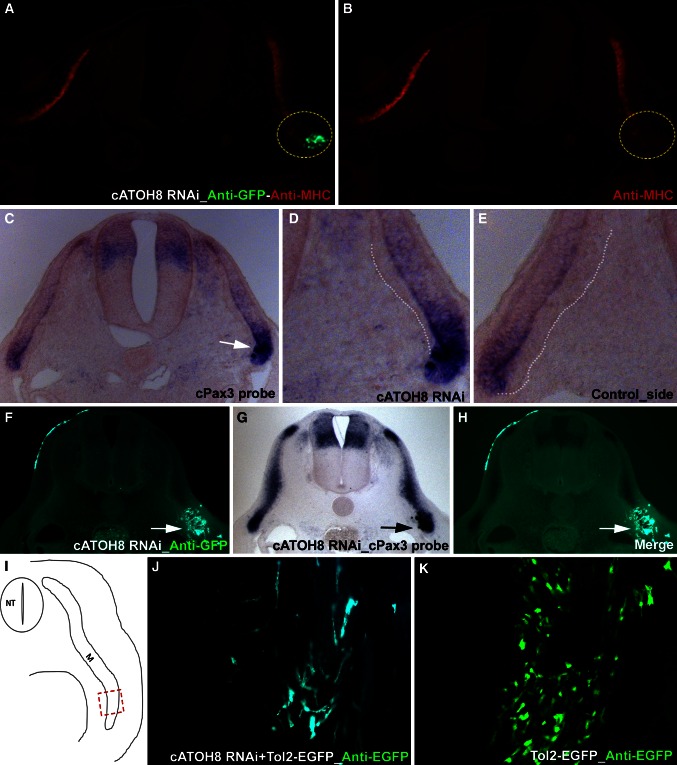
Silencing *ATOH8* in the somites affects muscle differentiation and cellular arrangement. The embryos electroporated with *ATOH8*-specific shRNA-EGFP constructs at the lateral somites were reincubated and processed for immunohistochemistry (myosin heavy chain—MHC). The targeted region is indicated by the EGFP expression (immunohistochemistry) (**a**). *ATOH8* silencing leads to the downregulation of MHC (*dotted circle* in **b**) at the targeted site (*dotted circle* in **a**). A section through the same embryo presented before in Fig. 2 **j**–**m** shows a clear upregulation of *Pax3* at the *ATOH8* shRNA electroporated region (**c**). The enlarged view of **c** shows defective hypaxial myotome formation after *ATOH8* knockdown (*white dotted line* in **d**). The normal myotome formation is shown on the control side (*white dotted line* in **e**). Anti-EGFP immunohistochemistry on the cross-sections of *ATOH8* shRNA electroporated and *Pax3* stained (in situ hybridization) embryo shows the co-localization of *Pax3* upregulation and EGFP expression, which indicates *ATOH8* shRNA (*white arrows* in **f**, **h**, *black arrow* in **g**). *ATOH8* RNAi (visualized here by co-electroporation of constructs in combination with the Tol2-EGFP expression system) in the hypaxial dermomyotome resulted in a remarkable distortion of hypaxial myotome development and caused patterning defects in the myofibers 4 days after electroporation (**j**). Electroporation of the Tol2 stable expression system alone did not show any distortion of myotome formation (**k**). *Red dashed rectangle* (**i**) indicates the hypaxial region in the embryo shown in **j** and **k**. *NT* neural tube, *M* myotome

To investigate the function of ATOH8 during chicken embryogenesis, we took advantage of the RNAi approach by targeting shRNA plasmids to specific regions of a chicken embryo. We targeted the lateral one-third of the dermomyotome and used EGFP signaling as an indicator of the electroporated region (Figs. 2, 3; Supplementary fig. 1). For our RNAi studies, we analyzed the effects of *ATOH8* silencing in the somites at trunk level (Fig. 1e, f). We first determined the inhibitory potential of the *ATOH8* shRNA constructs by showing a significant decrease in the expression of *ATOH8* corresponding to the EGFP signal (Fig. 2a–c). Next, we monitored the effect of silencing *ATOH8* expression. The resulting phenotype was that of a localized decrease in the expression of Myosin Heavy Chain (MHC), a marker of terminally differentiated muscle (Fig. 3a, b). We investigated the mechanism underlying the decrease in terminal muscle differentiation by examining the expression of early myogenic determination markers. Following *ATOH8* silencing, we also detected a downregulation of *Myf5* and *MyoD*, correlating with the electroporation site (Fig. 2d–i) and implicating a role for ATOH8 in skeletal myogenesis during embryogenesis. Interestingly, we found a significant upregulation of *Pax3* (Figs. 2k–m, 3c) in the targeted area (Fig. 2j). To check whether the *Pax3* upregulation is at the site of *cATOH8* silencing, we subjected the cross-sections of electroporated and *Pax3* stained (in situ hybridization) chicken embryos for immunohistochemistry analysis using anti-GFP antibody. The result confirmed that the *Pax3* overexpression is indeed at the site of *cATOH8* silencing (Fig. 3f–h). Moreover, at the location where the *ATOH8* silencing was performed, we observed a morphologically detectable defect in the formation of myotome from the hypaxial dermomyotome (Fig. 3c, d). On the other hand, normal myotome formation was observed on the untreated side (Fig. 3e), as well as in embryos electroporated with EGFP-only control constructs (Supplementary fig. 1 G, H). Supplementary table 1 provides an overview of the number of embryos analyzed for each gene. Control electroporation using the EGFP-only reporter control plasmid did not alter the normal expression of any of the genes analyzed (Supplementary fig. 1 A–H).

Co-electroporation of *ATOH8* shRNA constructs in combination with the Tol2-EGFP expression system (Sato et al. 2007; Kawakami 2007) enabled us to study the long-term effects of *ATOH8* silencing in the hypaxial dermomyotome by re-incubating the embryos for four more days. We observed a remarkably distorted patterning of VLL-derived cells normally destined to be myogenic progenitors. This was evidenced by the disordered cellular arrangement (Fig. 3j) compared to the parallel alignment of myofibers in the Tol2-EGFP-electroporated control specimens (Fig. 3k).

These results show that *ATOH8* is not only expressed in the hypaxial compartment of the myotome, but also in regions where somite-derived myogenic progenitors are transitioning from a progenitor to a determined myogenic myoblast state. Furthermore, we show that the decreasing *ATOH8* levels in somite-derived hypaxial dermomyotome result in defective hypaxial myotome formation and a decrease in the expression of both myogenic markers and MHC. Simultaneously, there was an increase in the expression of dermomyotome-derived progenitor cells.

### *ATOH8* expression in C2C12 myoblasts

**Fig. 4:**
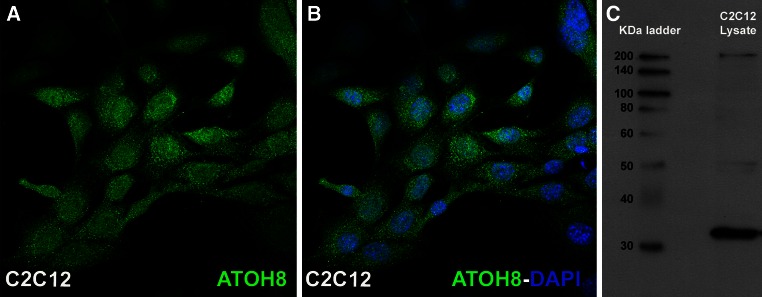
Expression of *ATOH8* in C2C12 myoblasts. C2C12 cells show a prominent expression of ATOH8 in the cytoplasm and in the nucleus (**a**, **b**). The nuclei are counterstained with DAPI (**b**). A Western blot of lysate from proliferating C2C12 cells gave positive bands for *ATOH8* expression (**c**)

Immunocytological staining was performed on C2C12 myoblasts grown on glass cover slips using ATOH8-specific primary antibodies. Confocal laser scanning microscopy of these cells showed a dotted pattern of endogenous ATOH8 expression in the cytoplasm, as well as in the nucleus (Fig. 4a, b). In order to confirm the presence of the ATOH8 protein, a Western blot was carried out using the cell extracts obtained from proliferating C2C12 myoblasts (Fig. 4c).

### *ATOH8* expression is regulated during myogenic differentiation

Using a mouse myoblast cell line real-time PCR-based system, the mRNA expression levels of endogenous *ATOH8* were quantified against the myogenic markers, *Myf5, Myogenin,* and *Pax7*. We exploited our finding that C2C12 cells express *ATOH8* and the fact that these cells can be forced to differentiate by changing the culture conditions, which has been well described in the literature (Lawson and Purslow 2000; Clemente et al. 2005).

The mRNA expression levels for each of the genes studied in proliferating C2C12 cells (cultured in normal growth media (T0)) were recorded. These were then considered as reference points to assess any alteration in the expression level of the respective genes during the C2C12 differentiation program.

**Fig. 5:**
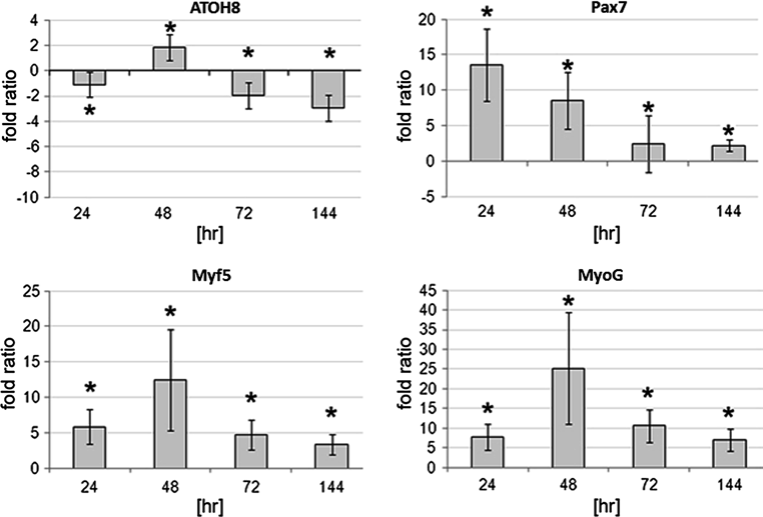
qRT-PCR of *ATOH8* and three myogenic markers isolated from C2C12 cells induced to differentiate revealed changes in steady state mRNA expression represented as fold ratio measured at 24, 48, 72, and 144 h (*T*). The fold ratios were compared to the baseline gene expression levels of proliferating C2C12 cells in growth medium (T0). Upon transfer into differentiation medium, a sharp increase in the levels of *ATOH8* was seen at 48 h, followed by a progressive decrease in the expression levels at T72 and T144. *Myf5* and *MyoD* expression levels also showed similar dynamics at all compared time points. *Pax7* expression levels, as expected, decreased progressively from T24 toward T144. A transient reduction in the number of *ATOH8* transcripts at T24 was also observed. Ratios were calculated using the REST software, with highly significant values denoted by *asterisks* (*p < 0.001)

As expected, we documented an upregulation in the myogenic differentiation genes, *Myf5* and *Myogenin,* following growth in differentiation culture (Fig. 5). In parallel, we observed a transient but significant increase in *ATOH8* mRNA on the second day in differentiation conditions, which is consistent with mRNA levels of *Myf5* and *Myogenin*. Following this, as the C2C12 cells continued their differentiation program, the number of *ATOH8* transcripts slowly taper off (6 days studied). A similar trend was observed for *Myf5* and *Myogenin* transcripts as well (Fig. 5). *Pax7* expression levels, as expected, decreased progressively from T24 (after one day) toward T144 (after 6 days). We also witnessed a transient reduction in *ATOH8* transcripts at T24, which could be attributed to the change in the culture media (growth medium to differentiation medium).

Thus, the onset of differentiation is accompanied by an immediate increase in *ATOH8* expression in C2C12 myoblasts in culture, pointing toward a function of ATOH8 in the transition from the committed to the differentiated state in myogenesis.

## Discussion

ATOH8 is a member of the Atonal bHLH transcription factor family. All members of the Atonal superfamily promote the differentiation of specific types of neurons (Yan and Wang 1998; Tomita et al. 2000; Hutcheson and Vetter 2001). In contrast to their well-characterized roles during neurogenesis, the function of the Atonal family is poorly understood in other developmental contexts. In this study, we show that the bHLH transcription factor ATOH8 is prominently expressed in the myotomal compartment during chicken embryogenesis. We demonstrate that reducing the levels of *ATOH8* results in a reduction in markers for myogenic determination and terminal differentiation. At the same time, we found an upregulation in the expression of the early premyogenic progenitor marker, *Pax3,* in the dermomyotome-derived premyogenic cells.

The myotome is the first primitive type of skeletal muscle to form in amniote embryos underneath the dermomyotome (DM). The initiation of myotome formation is attributed to the dorsomedial lip (DML), which is known to lay out the scaffold for the early epaxial myotome (Gros et al. 2004). The myotome is later populated by myoblasts, which enter the myotome in successive waves from all DM borders (Kahane et al. 2002; Hollway and Currie 2005; Yusuf and Brand-Saberi 2012).

The contribution of the DML to hypaxial myotome formation is variable along the craniocaudal axis of the developing embryo. The VLL at limb level disappears (Christ and Ordahl 1995) as a consequence of an epithelio-mesenchymal transition. This releases somitic precursors destined to form limb muscles and vessel endothelia, with minimal contribution to hypaxial myotome formation. The hypaxial myotome is mainly formed by the myogenic precursors from the medial border of the dermomyotome. In contrast, the hypaxial myotome receives no contributions from the medial dermomyotomal lip within the interlimb areas, as has been shown experimentally (Gros et al. 2004; Kahane et al. 2007).

Development of the hypaxial myotome is delayed in the flank region and is only formed after the medial (epaxial) myotome is already established. This delay of myotome maturation in the hypaxial domain is also reflected in the gene expression profile of the hypaxial myotome, in comparison with the epaxial myotome. The medial myotome expresses *MyoD,* as well as *Myf5,* in the chicken embryo, whereas the early hypaxial myotome only expresses *Myf5* and is devoid of *MyoD* transcripts. *MyoD* expression in the hypaxial myotome is only turned on when the medially located myotomal cells extend laterally toward the hypaxial myotome. This indicates that the delay in differentiation of the hypaxial myotome is abrogated by the medial pioneer myoblasts. Experimental manipulation of the expression of *MyoD* in the epaxial or hypaxial myotome leads to an abnormal myotome formation, fortifying the hypothesis that the medial pioneer myoblasts from the DML are also required for patterning the hypaxial myotome (Kahane et al. 2007). In our expression analysis, we identified some early expression of *ATOH8* in the hypaxial myotome from E2.5 (HH13) onwards, corresponding precisely to the hypaxial myotome prior to the expression of *MyoD* (as reported by Kahane and colleagues). Gradually, while progressing through the early developmental stages, the expression domain of *ATOH8* becomes extended medially, and at E3 (HH19), *ATOH8* is detectable in the epaxial myotome and DML of cranial somites. This spread of *ATOH8* expression occurs progressively along the craniocaudal embryonic axis. At HH13–14, *ATOH8* is expressed by the hypaxial myotome cells from the ventrolateral lip (VLL). These then exhibit a delayed differentiation program due to the lack of *MyoD* transcripts, as compared to their medial counterparts from the DML. It appears that ATOH8 may play a significant role in the differentiation of the hypaxial myotomes, which characteristically delay their differentiation, awaiting patterning cues from the medial myotomal cells. Silencing of *ATOH8* in the VLL perturbs the differentiation program of VLL progenitors, leading to the accumulation of myotome-destined muscle precursors at the targeting site. The later spread of *ATOH8* into the central and epaxial myotome during normal development is coherent with the entry of myoblasts from the rostral and caudal borders of the DM, as well as from the central DM (Gros et al. 2005). It is therefore tempting to believe that ATOH8 in the myotome may thus be required for the intercalating growth pattern suggested by the model of coherent myotomal growth (Kahane et al. 1998; Cinnamon et al. 1999). The silencing of *ATOH8* in zebrafish has been shown to disrupt myoseptum organization, affecting the typical chevron-shaped muscle arrangement (Yao et al. 2010).

The silencing of *ATOH8* in the lateral somites within the trunk region results in a decrease in *MyoD*, *Myf5,* and *MHC*, while *Pax3* is upregulated. As also suggested by our expression pattern analysis, *ATOH8* marks the hypaxial myotomal cells of the trunk. These cells are in a pre-differentiated state before the myoblasts, entering from the DML, have reached their lateral-most positions. The silencing of *ATOH8* in the lateral somites probably halts their progression toward differentiation. Additionally, the transfected group of predetermined muscle precursors failed to enter the myotome, remaining *Pax3*-positive. Moreover, interfering with the expression of *ATOH8* in the hypaxial myotome leads to distortion of the hypaxial cell arrangement. Parallel studies performed by our group where we overexpressed *ATOH8* in the ventrolateral lip of the dermomyotome, resulted in the notable upregulation of *MyoD,* accompanied with a distortion/forking of the hypaxial myotome (data not shown). We therefore believe that *ATOH8* expression is a necessary intermediate step between the undetermined progenitor status in the dermomyotome and the determined *MyoD* positive myoblast in the hypaxial myotome.

The data obtained in chicken embryos is in line with our in vitro analyses performed with C2C12 myoblasts, which are derived from murine adult skeletal muscle stem cells. The immunocytochemistry and Western blot analysis show that C2C12 cells also contain ATOH8 protein. Our C2C12 differentiation study followed by real-time PCR to analyze the expression levels of *ATOH8* showed that the *ATOH8* mRNA is expressed dynamically during the process of differentiation. Our results show that the expression of *ATOH8* was transiently upregulated after 48 h in differentiation medium, but significantly downregulated at all subsequently analyzed time points. The expression dynamics of *ATOH8* during this process also correlates with the same characteristic myogenic markers for adult myogenesis (*Myf5* and *MyoG*). As further proof of C2C12 myoblast differentiation, the expression level of Pax7, which is a skeletal muscle stem cell marker, became reduced concomitant with the progress of the differentiation program. Murine *ATOH8* (*MATH6*) expression in the hypaxial mouse myotome of embryonic stages 9.5 and 12.5 dpc has recently been reported (Rawnsley et al. 2013). Taken together, the data indicate that ATOH8 has a conserved, temporally restricted role in both embryonic and adult myogenesis, which may also span some species barriers.

Finally, our work shows that ATOH8 is required for the progression of development toward a differentiated state, in keeping with the roles played by other Atonal family members in their respective models. We thus propose that the expression of *ATOH8* is controlled by mechanisms that permit the development of a precursor population. The early detection of *ATOH8* transcripts in the hypaxial myotome of the trunk highlights yet another differing gene expression profile to add to the known list of markers in the myotome (Cheng et al. 2004; Ahmed et al. 2006). Furthermore, this study contributes to our current understanding of hypaxial muscle formation and may point toward specialized developmental mechanisms governing the formation of the hypaxial myotome as opposed to the epaxial myotome. Additional studies will be required to analyze the regulation of ATOH8 in the early myotome, more specifically, in the context of local signals.

## Materials and methods

### Chicken embryos and electroporation

Fertilized chicken eggs obtained from a local breeder were incubated at 37 °C and 80 % relative humidity for the required time to obtain stages 17–18HH. The embryo stages were determined according to Hamburger and Hamilton (Hamburger and Hamilton 1992). Embryos from HH10 to HH28 were killed and fixed in 4 % PFA for in situ hybridization. 2.5 ml albumin was removed using a syringe to lower the blastoderm, making the embryo accessible for the electroporation procedure. The upper side of each egg was windowed to visualize the embryos, followed by the partial removal of the extra embryonic membranes. The *ATOH8* shRNA constructs (2–3 μg/μl) were mixed with Fast Green solution (Sigma) at a ratio of 2:1 to ease the detection of injection site. For the co-electroporation of *ATOH8* shRNA constructs in combination with the Tol2-EGFP expression system, *ATOH8* shRNA constructs were mixed with constructs of *Tol2*-flanked CAGGS-EGFP (pT2 K-CAGGS-EGFP) and CAGGS-transposase (pCAGGS-T2TP) (Sato et al. 2007; Kawakami 2007). The constructs used in the Tol2 stable expression system were received from the laboratory of Koichi Kawakami. Tol2-EGFP-electroporated embryos were reincubated 4 days after electroporation. The DNA constructs were microinjected into the target somites and electroporated as previously described (Scaal et al. 2004; Dai et al. 2005). The electrodes were placed on each side of the microinjected embryo, and five square pulses of 30–55 V, 20 ms width were applied to each embryo. Upon passing current, the plasmid DNA with its negative charge was forced into the cells of tissues adjacent to the anode side. After 24-h reincubation, the EGFP expression in the transgenic embryos was visualized under fluorescence microscopy and photographed. The embryos were further sectioned using a Leica vibratome at a thickness of 35–60 μm. For permanent slides, sections were embedded in Aquatex from Merck.

### *ATOH8* shRNA constructs

We chose the plasmid-based RNAi strategy to downregulate *ATOH8* expression in chicken embryos. Four target sites were selected to prepare the shRNA constructs as described (Dai et al. 2005). Among them, three constructs containing target sequences, GTTGTCCAAACTGGCCATC, GAATAGCCTGTAACTATAT, and GAAGAGCTTCCAGCCAGTTA, were found to be effective, and a cocktail of both constructs was used for subsequent experiments.

### Whole-mount in situ hybridization

The EGFP expressing embryos were fixed overnight in 4 % paraformaldehyde at 4 °C. Separate mRNA riboprobes were used to detect the gene expression of *ATOH8*, *MyoD*, *Myf5*, and *Pax3*. Riboprobes were labeled with a digoxigenin RNA labeling kit from Boehringer, Mannheim, Germany. Whole-mount in situ hybridization was performed as described previously (Nieto et al. 1996) for normal untreated chicken embryos and for electroporated embryos. Double in situ hybridization was performed as previously described (Caprioli et al. 2002).

### Immunocytochemistry/immunohistochemistry

Sections of chicken embryo were fixed in 4 % paraformaldehyde at 4 °C overnight and were permeabilized with 0.5 % (v/v) Triton X-100 in PBS. C2C12 cells were grown on glass cover slips and fixed in the same way. Nonspecific antibody binding was blocked using 20 % (v/v) goat serum in PBS. Primary antibodies were applied overnight at 4 °C. Anti-ATOH8 (Sigma-Aldrich, 1:100), anti-MHC (DSHB, 1:200), and anti-GFP (Acris, 1:200) primary antibodies were used. Primary antibodies were visualized with fluorochrome-conjugated secondary antibodies (Molecular Probes) before mounting in DakoCytomation Faramount fluorescent mounting medium containing 100 ng/ml 4,6-diamidino-2-phenylindole (DAPI).

### Western blot

Cell lysate from proliferating C2C12 cells was boiled in 1 × Laemmli buffer for 5 min, separated by 10 % SDS–PAGE gel and blotted onto the nitrocellulose membrane. Blotted membranes were incubated with anti-ATOH8 primary antibody overnight. The blots were then incubated with HRP-conjugated secondary antibody for 1–2 h and the signal detected by SuperSignal West Pico Chemiluminescent Substrate (Pierce).

### Microscopy and imaging

After in situ hybridization, samples were observed and photographed using the Leica MZFLIII microscope and Leica DC 300F digital camera. Hybridized sections were observed and photographed using an Axioscope 20 (Zeiss) and a Leica DFC320 digital camera. Images of C2C12 myoblasts were captured using confocal laser scanning microscopy (Zeiss LSM 510 META) in the multi-tracking sequential mode set for the detection of AF-488 and DAPI with the appropriate emission bandpass filters.

### RNA preparation, reverse transcription, and quantitative real-time PCR of C2C12 cells

Total RNA was isolated from C2C12 cells using standard TRIzol method (Invitrogen). For real-time PCR, the first strand cDNA was synthesized using PrimeScript Reverse Transcriptase (TaKaRa, Japan). To confirm the successful cDNA synthesis, PCR was performed using primers designed against 18S RNA. SYBR Advantage qPCR mix (Clontech) was used to perform the real-time PCR. The primers were designed against mouse *ATOH8*, *Myogenin*, *Myf5,* and *Pax7* genes (sense *mATOH8* 5′-TCAGCTTCTCCGAGTGTGTG-3 antisense *mATOH8* 5′-TAGCCTGTGGCAGGTCACCT-3 sense *mMyogenin* 5′-GAAGCGCAGGCTCAGAAAGT-3′ antisense *mMyogenin* 5′-GATTGTGGGCGCTGTAGGGT-3′, sense *mMyf5* 5′-GACAGGGCTGTTACATTCAGG-3′ antisense *mMyf5* 5′-TGAGGGAACAGGTGGAGAAC-3′, sense *mPax7* 5′-GTCGGGTTCTGATTCCACAT-3′ antisense *mPax7* 5′- GCGAGAAGAAAGCCAAACAC-3′). Amplification reactions were performed in triplicates with the primer pairs producing single amplification products with a calculated Tms > 80 °C, which was verified by melting point analysis. Syber Green I reaction conditions were as recommended by the manufacturer (Clontech Mountain View, CA, USA) using the DNA Engine Opticon 2 Real-Time PCR Detection System (Bio-Rad Laboratories GmbH, München, Germany). The threshold cycle (*C*_t_) was defined at the point where the fluorescence signal reached a value of 0.01 above background during the exponential phase of the reaction. The average *C*_t_ values were used to calculate the ratios for all the genes studied using the equation procedure introduced by Pfaffl (Pfaffl et al. 2002). Ratios (*R*) represented the relative expression of a target gene in a sample:

$$R = (E_{\text{target}} ) \, C_{t}^{{({\text{reference - sample}})}} /(E_{\text{reference}} )c_{t}^{{({\text{reference - sample}})}}$$

Primer efficiencies (*E*) of the PCRs were determined directly from amplification curves using the DNA Engine Opticon 2 Real-Time PCR Detection System software. The *C*_t_ values for the reference gene *18s rRNA* were used to normalize mRNA levels of the samples. The upstream primer (5′-CATGGTGACCACGGGTGAC-3′) and the downstream primer (5′-TTCCTTGGATGTGGTAGCCG-3′) for *18s rRNA* were previously reported (Ray et al. 2005). The changes in the mRNA expression levels were calculated as ratios relative to the mRNA levels found at time point T0 of C2C12 differentiation. All experiments represented three independent sets of samples analyzed in triplicate. Statistical analysis was performed using the Relative Expression Software Tool (REST) software (Pfaffl et al. 2002) and the data expressed as expression ratios following the Pair Wise Fixed Reallocation Randomisation Test (Bustin et al. 2005).

## Electronic supplementary material

Below is the link to the electronic supplementary material.

**Supplementary** **Figure** **1**. The control EGFP reporter plasmid electroporations did not affect the normal expression pattern of any of the genes tested (A-H). During normal development (HH17 shown here), *ATOH8* expression is observable at the lateral lip of the dermomyotome (I). The enlarged view of *ATOH8* expression (marked by dashed rectangle in I) at the lateral dermomyotomal lip is shown in J (black arrows in J). (JPEG 1128 kb)

**Supplementary** **Table** **1**. States the number of embryos electroporated with the *ATOH8*-specific shRNA-EGFP construct and analyzed using in situ hybridization for the expression of *ATOH8*, *Myf5*, *MyoD* and *Pax3.* The percentages of observed effects are included. (JPEG 39 kb)
