# Supplementary figures and images for: ATOH8, a regulator of skeletal myogenesis in the hypaxial myotome of the trunk
Source: Histochem Cell Biol. 2013 Nov 2;141(3):289–300. doi: 10.1007/s00418-013-1155-0 (PMC3935115; doi:10.1007/s00418-013-1155-0)

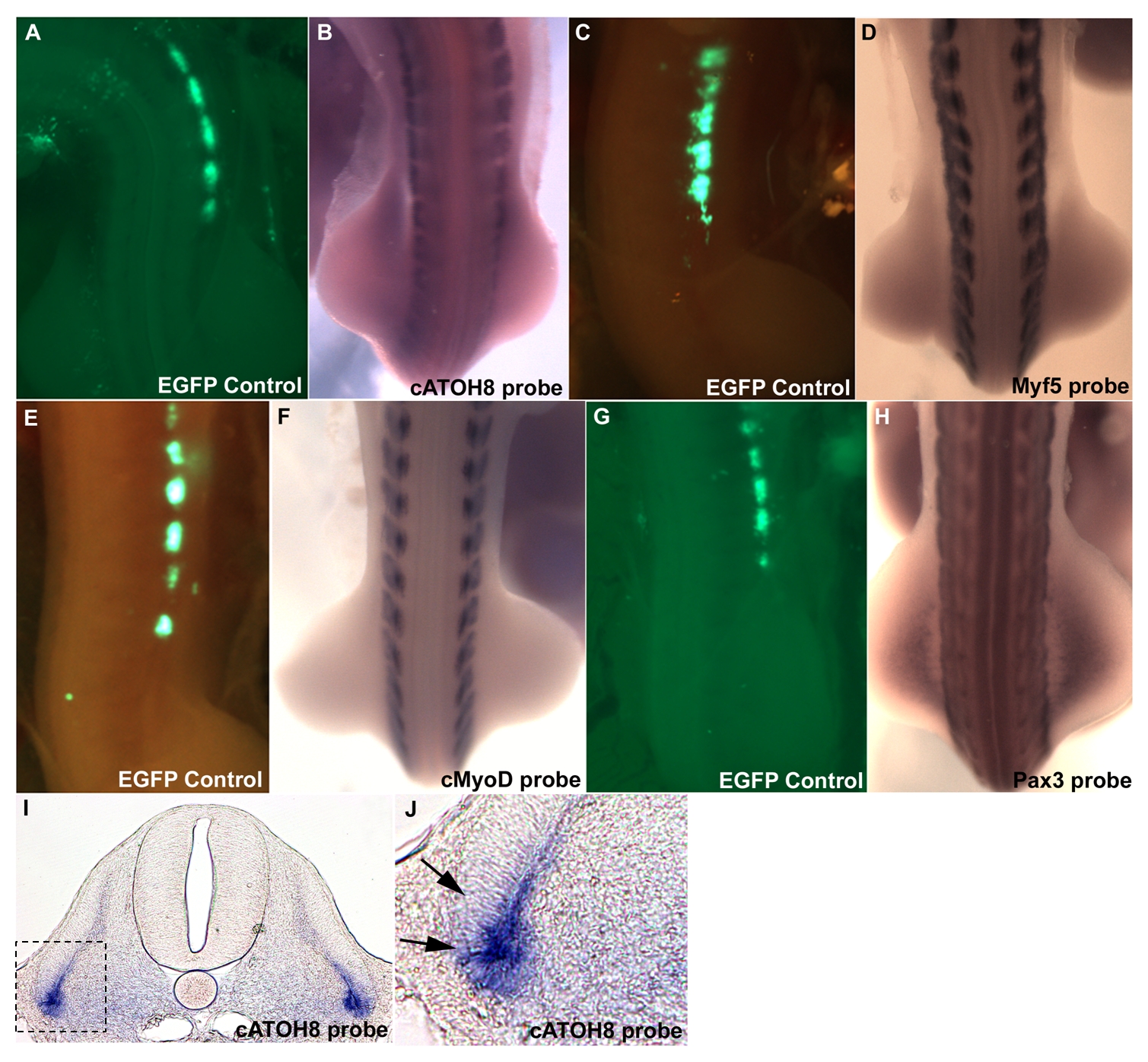

Supplement: Supplementary file 1 — Supplementary Figure 1. The control EGFP reporter plasmid electroporations did not affect the normal expression pattern of any of the genes tested (A-H). During normal development (HH17 shown here), ATOH8 expression is observable at the lateral lip of the dermomyotome (I). The enlarged view of ATOH8 expression (marked by dashed rectangle in I) at the lateral dermomyotomal lip is shown in J (black arrows in J). (JPEG 1128 kb) [file 418_2013_1155_MOESM1_ESM.jpg]

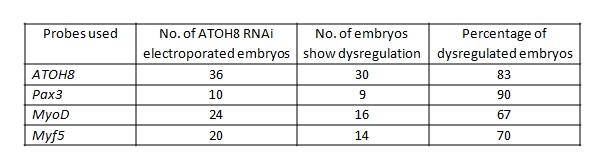

Supplement: Supplementary file 2 — Supplementary Table 1. States the number of embryos electroporated with the ATOH8-specific shRNA-EGFP construct and analyzed using in situ hybridization for the expression of ATOH8, Myf5, MyoD and Pax3. The percentages of observed effects are included. (JPEG 39 kb) [file 418_2013_1155_MOESM2_ESM.jpg]
